# A review of virulent Newcastle disease viruses in the United States and the role of wild birds in viral persistence and spread

**DOI:** 10.1186/s13567-017-0475-9

**Published:** 2017-10-26

**Authors:** Vienna R. Brown, Sarah N. Bevins

**Affiliations:** 1 Oak Ridge Institute for Science and Education (ORISE) supported by the U.S. Department of Homeland Security (DHS), Science and Technology Directorate (S&T), Chemical and Biological Defense Division (CBD), Oak Ridge, TN USA; 2 United States Department of Agriculture, Animal and Plant Health Inspection Service Wildlife Services, National Wildlife Research Center, Fort Collins, CO USA

## Abstract

Newcastle disease is caused by virulent strains of Newcastle disease virus (NDV), which causes substantial morbidity and mortality events worldwide in poultry. The virus strains can be differentiated as lentogenic, mesogenic, or velogenic based on a mean death time in chicken embryos. Currently, velogenic strains of NDV are not endemic in United States domestic poultry; however, these strains are present in other countries and are occasionally detected in wild birds in the U.S. A viral introduction into domestic poultry could have severe economic consequences due to the loss of production from sick and dying birds, the cost of control measures such as depopulation and disinfection measures, and the trade restrictions that would likely be imposed as a result of an outbreak. Due to the disease-free status of the U.S. and the high cost of a potential viral incursion to the poultry industry, a qualitative risk analysis was performed to evaluate the vulnerabilities of the U.S. against the introduction of virulent strains of NDV. The most likely routes of virus introduction are explored and data gathered by several federal agencies is provided. Recommendations are ultimately provided for data that would be useful to further understand NDV on the landscape and to utilize all existing sampling opportunities to begin to comprehend viral movement and further characterize the risk of NDV introduction into the U.S.

**Electronic supplementary material:**

The online version of this article (doi:10.1186/s13567-017-0475-9) contains supplementary material, which is available to authorized users.

## Introduction

Newcastle disease (ND) occurs in poultry and can have devastating economic effects on global domestic poultry production. Newcastle disease virus (NDV) was previously synonymous with avian paramyxovirus type 1 (APMV-1); however, due to changes in taxonomy is now referred to as avian avulavirus [1]. Avian avulavirus and NDV are used interchangeably in this manuscript. NDV can be distinguished into three distinct pathotypes based on mean death time in chicken embryos, lentogenic (40–60 h), mesogenic (60–90 h), and velogenic (90–150 h) [2]. The amino acid sequence at the fusion cleavage site is related to virulence as specific sequences can be cleaved systemically as compared to others which can only be cleaved in specific host tissues leading to a local infection. In this way, all velogenic viruses are virulent but not all virulent strains are velogenic. This disease is listed by the World Health Organisation for Animals (OIE) as vitally important for avian species and products and virus detection in a specific geographical location often leads to trade restrictions and embargos [3]. Virulent Newcastle disease is listed as a Tier 1 USDA Select Agent as it is a pathogen of national concern and a significant threat to animal agriculture in the U.S. Only mesogenic and velogenic viruses found in poultry species, require mandatory reporting to the OIE. Virulent strains, typically mesogens and velogens, of avian avulavirus are endemic in the majority of Asia, Africa, and the Middle East, as well as parts of Central and South America in domestic poultry species [4]. In the United States, virulent strains are present in wild pigeons and cormorants, but domestic poultry are free of these virus forms. Detection of velogenic NDV in domestic poultry could have devastating financial effects on the industry as well as the nation at large. The last outbreak of velogenic NDV in the United States occurred in 2002–2003 in California, Nevada, Arizona, and Texas in domestic poultry (confined to backyard flocks in the latter three states, but did spillover into a commercial operation in California) resulting in the culling of 3.16 million birds at a cost of $121 million [5]. Low virulence strains of NDV occur throughout the world in both domestic and wild bird species. Understanding the importance wild bird species may play in the maintenance and transmission of this virus in addition to their proximity to domestic poultry production is needed in order to minimize the risk of infection. This manuscript further describes the zoonotic potential for this disease, as well as available vaccines, and commonly used diagnostic tools. Furthermore, a risk assessment is described and recommendations are provided.

### Description of the virus and infection kinetics

A negative sense, single stranded RNA virus is responsible for Newcastle disease, also referred to as avian avulavirus, which is capable of infecting more than 250 species of birds [4]. This virus is endemic in many parts of the world and has been known to cause epizootic outbreaks in domestic poultry on six of the seven continents [6]. The genome contains 6 genes and their corresponding structural proteins: (1) a nucleocapsid protein (NP), (2) a phosphoprotein (P), (3) a matrix protein (M), (4) a fusion protein (F), (5) a hemagglutinin-neuraminidase protein (HN), and (6) a RNA polymerase (L) [7, 8].

The molecular basis for the variation in virulence has been determined and is attributed to amino acids at the cleavage site on the fusion protein, which mediates cell-virus and cell–cell interaction and fusion [9]. Three or more arginine (R) or lysine (K) residues starting at position 113 and a phenylalanine (F) at position 117 are found in all virulent strains of the virus. The precursor fusion protein, F0, must be cleaved to proteins F1 and F2 by host cell proteases in order for infection to begin via cell fusion and the activation of hemolytic properties; the proteases recognize the specific amino acid motif at the F protein cleavage site [3, 10]. Viruses with a virulent cleavage site can be cleaved by proteases found in nearly every cell in the body allowing for a systemic infection to occur and extensive viral replication; however, if the F protein cleavage site does not contain that specific amino acid motif, cleavage can only be mediated by trypsin and trypsin-like enzymes found in the respiratory and intestinal tracts which leads to restricted host site replication [11, 12]. The hemagglutinin-neuraminidase protein also plays an instrumental role in tissue tropism and virulence of NDV by promoting the fusion activity of the F protein, facilitating host cell penetration, and removing sialic acid from progeny virus to prevent self-agglutination [9]. Enveloped viruses, including NDV, have been found to enter host cells via direct fusion methods in which the viral envelope fuses with the plasma membrane of the host cell or a receptor-mediated endocytosis mechanism in which the virus binds to a specific receptor on the host cell surface and membrane fusion results in translocation of the nucleocapsid into the cytoplasm of the host cell [13, 14].

It is important to note that avian avulaviruses has been used as a therapeutic agent in the treatment of human cancer. Replication-competent oncolytic viruses, such as NDV, have a pronounced anti-tumoral effect by both local and systemic inoculation routes [15, 16]. NDV has been found to efficiently and selectively replicate within and destroy tumor cells, but not normal cells, and intra-lesion inoculation in an athymic mouse model has resulted in complete tumor regression [17]. Avian avulaviruses can stably express foreign genes and as such, experimental trails have been conducted where the gene for IL-2 is incorporated [18]. NDV in combination with IL-2 was found to be especially T cell stimulatory which increases the anti-tumor capacity. A strong type 1 interferon response is induced in non-permissive species, such as humans, which results in the anti-neoplastic and immune stimulatory properties observed with NDV infection [19].

### Transmission and clinical signs

NDV is primarily transmitted via inhalation or ingestion of virus shed in feces and respiratory secretions by infected birds for variable lengths of time [3, 4, 20]. Some virus isolates have been found to be transmitted through the egg to the hatching chick [21]. Furthermore, virus has been found to be present in all parts of the carcass and is able to persist for many months on both chicken skin and bone marrow if kept at refrigerated temperatures [4]. The virus is relatively stable outside of a host and in the environment, thus making fomite transmission a possibility. Infectious virus has been found to survive 7 days in the summer, 14 days during the spring, and 30 days in the winter in poultry houses that were contaminated by infected birds [22]. An older study demonstrated the duration of infectious virus using various materials and temperatures which suggests that NDV is a highly stable virus on multiple types of materials and at various temperatures [23] and confirms the concern of fomites as a vehicle for virus introduction or during an outbreak.

The incubation period is typically 2–15 days post-exposure. Gallinaceous birds (chickens, turkeys, grouse, pheasants, and partridges) shed infectious virus for up to 1–2 weeks following infection; however, psittacine birds (parrots, parakeets, and macaws) have demonstrated the capacity to shed infectious virus for several months to 1 year following infection, primarily via respiratory secretions and feces [24, 25]. It is likely that the mortality rate for chicks born with virulent NDV infections would be very high, especially as maternal antibody levels waned.

The clinical manifestation of a velogenic strain of NDV, typically results in a primarily intestinal infection characterized by hemorrhagic lesions found in the intestines of dead birds (termed viscerotropic velogenic) or a predominantly respiratory and neurological infection (referred to as neurotropic velogenic) [6]. Neurological signs are also often observed with velogenic infections, especially in birds with partial immunity who often go on to develop a chronic infection, and the signs typically include tremors, ataxia, torticollis, and paresis or paralysis of the wings or legs which develop several days post-infection [4]. Both wild and domestic species can develop similar neurotropic signs. Mesogenic viruses can cause clinical disease which typically include respiratory and neurological signs but the infection is self-limiting, and mortality is rare in older birds unless there are secondary bacterial infections [4]. Lesions associated with both velogenic and mesogenic strains of NDV infection are most often detected in the central nervous system (CNS), alimentary tract, renal system, or respiratory tract and viruses virulent in wild bird populations tend to affect the CNS or kidneys or to cause systemic disease that results in rapid mortality in the absence of recognizable gross or histopathological lesions [3].

### Geographical distribution

Newcastle disease is believed to have first been reported in 1926 in both Newcastle-on-Tyne, England and the island of Java, which is part of current day Indonesia; however, there is evidence of prior emergence based on literature in which outbreaks of disease similar to NDV were reported [26]. The virus is now endemic or causes epizootic events on a global scale [6]. Avian avulavirus strains are phylogenetically classified into class I and class II and class II are further differentiated into separate genotypes based on genetic and geographic variations [27]. Pigeon paramyxovirus type 1 (PPMV-1) is a type of avian avulavirus and is listed under class II, genotype VIb viruses (Additional file 1).

The viral presence in both Mexico and Canada was specifically investigated as their disease status influences risk of viral introduction into the United States based on close proximity. Virulent strains of NDV are endemic in Mexico, isolates detected typically belong to the class II, genotype V viruses [28]. Sampling of free-ranging wild birds, captive wild birds, and domestic poultry demonstrate continued circulation and evolution of these viruses [29]. The Canadian Food Inspection Agency states that virulent strains of NDV are exotic to poultry in Canada; however, similar to the U.S., wild birds in Canada have been found to harbor these viruses [30].

## Wild bird reservoir species

Birds of the Columbidae family (pigeons and doves) and double-crested cormorants (*Phalacrocorax auritus*) have been implicated as reservoir species for virulent strains of NDV in North America [31]. Sampling of mute swans (*Cygnus olor*) in the Great Lakes region and along the Atlantic coast of the United States resulted in the detection of live virus and NDV antibodies in 8.7 and 60% of birds, respectively [32]. This suggests that mute swans are regularly exposed to avian avulaviruses and may contribute to viral maintenance in the environment. Kim et al. sampled waterfowl and shorebirds from eight states within the U.S. and compared them to samples from live bird markets collected between 2005 and 2006 and found genetically related viruses [33]. Research on NDV in wild ducks, gulls, and shorebirds found novel viral diversity, but no fusion gene sequences associated with high pathogenicity in poultry [34]. These findings indicate that viral transmission may occur between wild birds and poultry, but virulent strains are likely not reservoired by the majority of wild birds, with pigeons, doves, and double-crested cormorants being the exception.

Interestingly, evaluation of wild birds on four continents between 1997 and 2014 resulted in the repeated isolation of vaccine-derived NDV [35]. These vaccine-derived isolates were found in 17 species with the highest frequency detected in Columbiformes and Anseriformes. Not surprisingly, the vaccine strain viruses found corresponded to those that are most widely utilized as vaccines—La Sota and B1. These findings are troubling as passaging through wild bird species may provide selective pressures that could lead to antigenic drift or an increase in virulence. While concerning, there is no documented case of vaccine strains recombining with wild type strains in wild birds; however, research to date on NDV viral dynamics in wild birds has been limited and more thorough exploration could better determine if this is occurring. There is, however, one case of a low virulence wild bird strain from class II genotype I NDV mutating to a virulent strain after circulating for many months in thousands of chickens that lead to the 1998 ND outbreak in Australia [36].

### Double-crested cormorants

The first documented mortality event associated with NDV in wild avian species, young double-crested cormorants, was first reported in Canada in 1975. Since then, several other outbreaks have occurred in the 1990s and 2000s in both the U.S. and Canada and sero-prevalence in adult birds is high [37–39]. A study conducted on breeding and wintering grounds in the U.S. and Canada between 2009 and 2011, found the average antibody prevalence in adult birds, across all years and locations, to be 85.2% [31]. Live, infectious NDV isolates were isolated from 6 chicks, half of which exhibited clinical signs typical of a NDV infection. Furthermore, virulent NDV from outbreaks in double-crested cormorants have been implicated in causing mortality in pelicans, gulls, and other shorebirds [40, 41]. Findings suggest that double-crested cormorants are regularly exposed to avian avulavirus isolates and that these infections are not necessarily lethal. They may play an important role in maintaining and amplifying the virus in nature. Double-crested cormorants inhabit a large portion of North America between their breeding and wintering grounds and this nearly ubiquitous distribution underlines their important role in the spread of NDV [42].

### Columbidae birds

Numerous species of wild and domestic pigeons and doves have been implicated in the maintenance and transmission of pigeon paramyxovirus serotype 1 (PPMV-1) which is a class II, genotype VIb avian avulavirus that is host-adapted to pigeons and other Columbiform birds [27, 43]. These viruses are virulent variants of avian avulavirus that have circulated in pigeons and are adapted to this species. These viruses are considered to be panzootic despite having originated in the Middle East and being detected as early as the 1980s [43]. Columbiform birds are the primary host species; however, spillover events into domestic poultry have been reported and serial passages through embyronated chicken eggs or domestic poultry species appears to result in selection for more virulent traits [44, 45]. Clinical signs associated with PPMV-1 infection in layer hens is typically limited to a sharp reduction in egg production, mis-shapen eggs, or soft shell eggs [43]. Domestic chickens were experimentally inoculated with various isolates of PPMV-1 viruses intramuscularly and gross and histologic lesions were observed as well as morbidity [44]. Overt clinical disease was rare and none of the infected chickens succumbed to disease during the follow-up period (14 days post-infection). Immunohistochemistry determined that the heart and brain were the primarily affected tissues. Sampling in 2013 detected seropositivity in 11.7% of Columbiformes evaluated which included Eurasian collared doves (*Streptopelia decaocto*), rock doves (*Columba livia*), and zebra doves (*Geopelia striata*) [46]. All of the viruses isolated from this study were found in rock doves and found to be virulent strains furthering the assertion that rock doves are associated with PPMV-1 virus maintenance in the United States. They are found uniformly throughout the U.S. [47].

## Domestic poultry

### Geographical distribution

**Figure 1:**
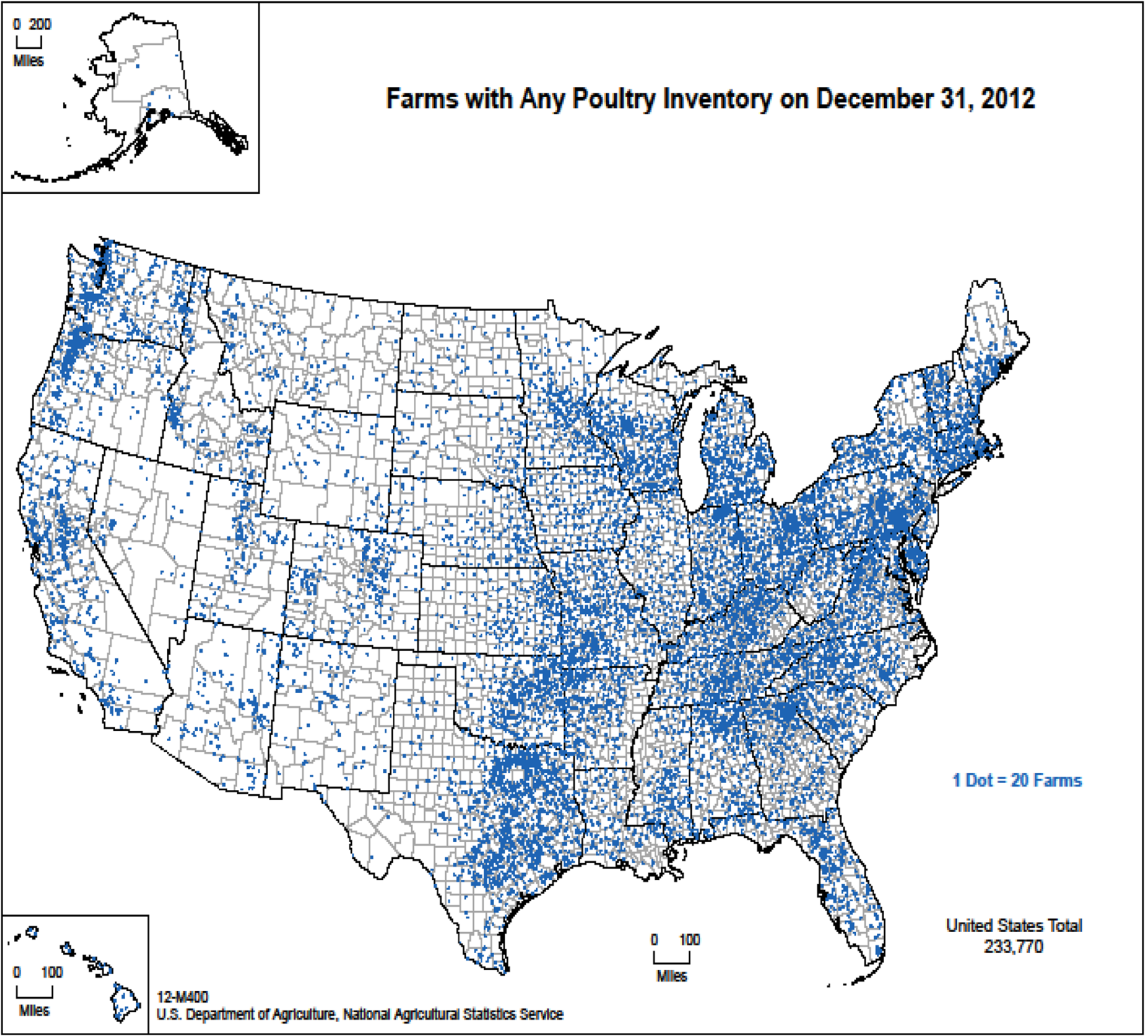
**Distribution of poultry production within the United States, 2012.** Figure from the United States Department of Agriculture, National Agricultural Statistics Service (2015), used with permission—[51].

The United States poultry industry generates $48 billion in revenue and is comprised primarily of broiler chickens (8.5 billion birds), layer hens (299 million birds), and turkeys (238 million birds) [48]. Iowa, Indiana, Ohio, Pennsylvania, and Texas are the top five layer producing states and are responsible for 52% of all the eggs produced within the United States [49]; whereas top broiler producing states include Georgia, Arkansas, Alabama, North Carolina, and Mississippi [50]. Figure 1 depicts poultry inventory across the entire U.S., irrespective of bird or farm type.

Domestic poultry production certainly overlaps with the geographical distribution of the two primary wild avian reservoir species. The last detectable outbreak of virulent NDV in domestic poultry in the U.S. occurred in 2002–2003, starting in illegally imported game fowl [52]. This outbreak initiated in southern California before moving into Nevada, Arizona, and ultimately, Texas; with infections being confined to backyard flocks in all states but California where the virus did spillover into commercial operations [53]. Phylogenetic analysis of the virus showed that the strains were very similar to those circulating in Mexico and Honduras and that the California, Nevada, and Arizona viruses were a result of a single point introduction of the virus.

### Commercial domestic poultry management

Exact information and specific details related to poultry production in the United States is particularly difficult to acquire and much of it is kept as proprietary data. A huge majority (likely > 98%) of domestic poultry are intensively managed with nearly 100% of breeding stock and 100% of large, commercial poultry operations existing in this type of system. In recent years, there have been changes in labeling (ex: cage free, free range) and subsequently, management practices have been modified to reflect these production systems. However, these “specialty” production systems compose a tiny fraction of the overall production of meat and eggs in the U.S.

The National Poultry Improvement Plan (NPIP) is a collaborative program between industry and state and federal governments that was originally established to abolish pullorum disease and today serves to enhance international trade and prevent the introduction of pests and diseases that would be costly to the poultry industry and agricultural resources. This program includes commercial poultry, turkeys, waterfowl, exhibition poultry, backyard poultry, and game birds. Fourteen biosecurity principles were drafted and presented at the Biennial Conference for NPIP, which occurred August 30^th^ to September 1^st^, 2016 at which time they were amended and ultimately, voted on. All of the proposed principles were passed in either their exact drafted form or a slightly amended version. Commercial poultry operations in all participating states (which includes all U.S. states, except Hawaii) that desire to transport birds across state lines or engage in international export are then responsible for implementing the principles on their premise. Principle #14 describes the auditing process, but briefly, auditing is performed by the Official State Agency to ensure compliance at least once every 2 years.

Information regarding the majority of domestic poultry operations and the likely impending biosecurity regulations from the NPIP have been described to provide information on the management of commercial poultry and insight relevant to biosecurity concerns and practices. This intensive management and attention to high risk routes of contamination offers a vantage point related to preventing Newcastle disease virus or detecting and confining an outbreak very quickly, should a velogenic strain be introduced. Biosecurity must be enacted along with vaccination protocols to enhance the efficacy of the control program.

### Common vaccination protocols in the United States for commercial poultry operations

The domestic poultry industry is not mandated to provide vaccinations against specific pathogens by any governing body; thus, each producer can determine what vaccines they would like to administer. This is typically based on geographical location, life span, perceived risks, and other components; however, industry experts believe the prevalence of NDV vaccination to be close to 100% for intensively managed domestic poultry in the United States. Long-lived birds, specifically broiler breeders and layers, are usually vaccinated multiple times for NDV whereas shorter lived birds typically receive one vaccine, either in ovo or at 1 day of age in the hatchery (Personal communication, U.S. Poultry and Egg Association, 2016). The in ovo vaccine is a recombinant herpesvirus of turkeys/Newcastle disease virus vaccine, whereas the vaccine administered to day-old birds is typically a live NDV vaccine. Broiler breeders often receive multiple rounds of vaccinations for NDV of both live and inactivated vaccines in order to provide the hatchling with sufficient levels of maternal antibodies. Layers tend to be vaccinated less intensively and typically receive a live vaccine as it is simpler to administer (often as a spray or in drinking water). The NDV vaccines are primarily designed to prevent endemic low virulence strains that cause mild respiratory signs from infecting domestic poultry which, if unresolved at the time of slaughter, often result in carcass condemnation which causes losses for producers. Industry insiders believe the U.S. domestic poultry population is still, despite high levels of vaccination, highly susceptible to velogenic strains of NDV in the event of viral introduction.

### Domestic backyard poultry management

The level of management provided to backyard poultry flocks is extremely variable, although it is very common that these flocks are more extensively managed as compared to commercial poultry and likely < 10% of backyard poultry are vaccinated in any capacity (Personal communication, Merial, 2016). While backyard poultry flocks are a small fraction of U.S. poultry production, they are of greater concern to be exposed to virulent avian avulavirus because of the lack of vaccination and biosecurity. They may serve as amplification hosts which increases the probability that virulent NDV could spill over into commercial poultry flocks due to large amounts of circulating virus. As an example, Europe is considered free of virulent NDV in commercial poultry; however, outbreaks in backyard birds occurs periodically [54, 55]. Given the breadth of exposure of backyard poultry to wild birds and environmental factors, surveillance efforts in these domestic birds may be useful to gather data on circulating viruses and their pathogenicity.

## Zoonotic potential

Avian avulaviruses are capable of infecting humans and infection typically results in conjunctivitis and/or influenza-like symptoms, including fever, headache, and malaise [4]. Exposure to a large amount of virus is necessary, thus human infections are most common in individuals working on poultry farms or in slaughterhouses. There has been one documented instance in which a 42-year old man with a history of non-Hodgkin’s lymphoma developed a lethal pneumonia following a peripheral blood stem cell transplant [56]. Genomic sequencing demonstrated that the NDV strain found in the deceased patient was likely an isolate of urban-dwelling doves and pigeons. The patient was not known to have any contact with avian species and the authors hypothesize that he was exposed in his urban neighborhood either through direct contact or aerosolized bird feces containing infectious virus.

Despite the ability for avian avulaviruses to infect humans this pathogen should be considered a low priority for public health agencies because of the high infectious dose and the mild clinical symptoms observed in immune-competent individuals. Fomite transmission of NDV is a documented route of spread due to robust viral stability; thus, special precautions should be taken for individuals that work closely with domestic poultry and are immune-compromised or for workers that co-habitate with an individual that is immune-compromised [3].

## Vaccines

Multiple vaccination regimens are used throughout the world for domestic poultry and are fairly efficacious in preventing clinical disease, although there is no sterilizing immunity and vaccinated birds may be infected and shed virulent NDV without becoming ill [57, 58]. All NDV strains are of the same serotype, thus nearly any strain can be used in a vaccine because of similar antigenic properties resulting in a fairly uniform immune response irrespective of the vaccine strain [59]. However, further work has demonstrated that vaccine strains that are homologous with the challenge virus or field strains expected to infect the birds in a given region, are able to substantially reduce oral shedding as compared to heterologous vaccines; furthermore, the evolution of virulent strains of NDV may be facilitated by the interaction of vaccine strains and endemically circulating viruses that have large phylogenetic or antigenic variation in comparison to the vaccine strain [6, 60].

Two vaccination methods are predominant: inactivated and live vaccines [61]. Inactivated vaccinations are considerably more expensive as compared to live vaccines due to the need to handle individual birds and provide an injection, either intramuscularly or subcutaneously [62]. Live vaccines are often provided in mass either via a spray or drinking water. Both live and inactivated vaccines are produced in specific-pathogen-free (SPF) embryonated eggs [61].

A wide variety of vaccines and vaccination protocols are utilized across the globe depending on endemic strains, domestic poultry production practices, access to a cold chain, and many other factors. The B1 and LaSota strains are lentogenic viruses that are commonly used in vaccination schemes, often serially [59, 63]. B1 is a very mild strain and is often administered initially, followed by a booster with a slightly more pathogenic vaccine strain, such as LaSota. Initial vaccination typically occurs either at 2–4 weeks of age (after maternal antibody has waned) or at 1 day of age which results in an active infection that will persist until maternal antibody has waned [62]. These birds are then re-vaccinated at 2–4 weeks of age.

The mass inoculation strategy of live (sometimes attenuated) vaccines has resulted in concern about the potential for reversion to virulence of attenuated strains or the leaching of these strains into the environment [6, 62]. In fact, Miller et al. articulate three factors that contribute to the risk of an outbreak: (1) only a few nucleotide changes are needed on the fusion gene to convert a low virulence virus to a strain of high virulence, (2) the low virulence viruses are endemic nearly universally and large, highly mobile reservoirs are capable of moving these viruses around the globe, (3) billions of (mostly live) vaccines are administered annually, often in a spray or drinking water, which likely leads to environmental contamination. Importantly, vaccinated poultry have been implicated as the reservoir for virulent strains of NDV as a result of the ability to become infected with virulent strains following vaccination and shed infectious virus in the absence of clinical disease [6]. This topic is still widely discussed as some believe shedding of infectious virus is resultant from incomplete or non-uniform mass vaccination methods; however, some evidence does exist to suggest that vaccinated birds may serve as reservoirs for virulent NDV [64]. Furthermore, vaccination may be providing selective pressure that favors the evolution of variant forms of NDV, especially driven by the genetic homogeneity of hosts, production practices (high density), and intensive and imprecise vaccination protocols.

Additionally, a recent review of NDV vaccines by Dimitrov et al. asserts that new vaccine concepts are needed as current vaccination protocols are insufficient to quell disease under various environmental conditions. Challenges identified are specifically related to uneven vaccine application under mass vaccine administration techniques, difficulty vaccinating free-roaming birds, especially those of varying ages, obstacles maintaining the cold chain, and pre-existing antibodies which can neutralize the vaccine and reduce efficacy [65].

## Diagnostics

A wide variety of diagnostic tools have been developed to determine pathogen identity. These methods are often used serially. Samples collected from sick and dying birds can be inoculated into embryonated eggs and further differentiation can be undertaken. Mean death time (MDT) is a frequently used diagnostic which involves inoculating samples into embryonated eggs and determining the time in hours required to kill the chicken embryo [3]. The intracerebral pathogenicity index (ICPI) and the intravenous pathogenicity index (IVPI) involve the weighted scoring of clinical signs following an intracerebral or intravenous injection, respectively, in day old chicks. ICPI is the test of choice for NDV whereas IVPI is used only occasionally and never for official purposes. The gold standard for NDV diagnostics is virus isolation followed by hemagglutination and hemagglutination inhibition. Table 1 depicts the MDT, ICPI, and IVPI associated with lentogenic, mesogenic, and velogenic viruses. These values are derived from Alexander 1998 but it should be noted that exceptions exist [66]. Further pathotyping often occurs when live virus is isolated in order to determine strain virulence via the evaluation of the amino acid residues at the fusion site.

**Table 1:** **Summary of diagnostic parameters associated with avian avulavirus**

Diagnostic tool	Velogenic strains	Mesogenic strains	Lentogenic strains
Mean death time	< 60 h	60–90 h	> 90 h
Intracerebral pathogenicity index	1.5–2	1–1.5	0.0–0.5
Intravenous pathogenicity index	2.0–3.0	0–0.5	0.0

The costs and challenges associated with obtaining SPF eggs and day-old chicks make real-time RT-PCR methods an attractive alternative [67]. From a serological perspective, a hemagglutinin inhibition (HI) assay or an enzyme-linked immunosorbent assay (ELISA) can be used to evaluate the presence of NDV-specific antibodies in the host [6]. Both the HI and ELISA diagnostics can be used to detect antigen or antibody. Serological diagnostics do not allow for the differentiation of antibodies resultant from exposure to a vaccine strain as compared to a virulent NDV isolate; therefore, they are not tremendously efficacious for the determination of NDV as the cause of a specific outbreak because of rampant vaccination. Many commercial operations closely monitor the serology of a flock at the time of slaughter, often using an ELISA (Personal communication, Merial, 2016). Based on years of data, a normal range is determined for the antibody titer for NDV and anything that exceeds that value is subject to further testing and more intensive evaluation. This monitoring helps identify an abnormality very quickly and mobilize a response in the event of an introduction.

## Emergency preparedness for the United States

A viral incursion of NDV into the U.S. could likely cause severe morbidity and mortality in the domestic poultry industry in addition to enormous economic losses primarily associated with trade restrictions. The Newcastle Disease Response Plan, The Red Book was created in 2014 by USDA APHIS Veterinary Services and provides strategic guidance for government officials and first responders in the event of a NDV outbreak. The manual also provides current policy information and a strategic framework for the control and eradication of NDV in the event of a viral incursion. Furthermore, the FAD Prep Document provides information about the Newcastle disease virus such that responders and stakeholders can have a common understanding of the pathogen etiology.

## Risk analysis

Globalization, international trade, and migratory wild avian species of questionable NDV infection status increase the likelihood of virulent avian avulavirus introduction. A number of routes are of concern, including a point introduction via transportation of live birds or poultry products, viral persistence on fomites, or contaminated poultry food, water, or supplies. Because of the prevalence of wild avian species that are known to carry virulent strains of NDV it is also possible that an outbreak is initiated by a spillover event in which a virulent strain of NDV from double-crested cormorants, rock doves, or alternative wild bird types are transmitted to domestic poultry. Additionally, backyard domestic poultry pose a risk of viral transmission and amplification due to their exposure to wild birds and other environmental factors. Finally, because of the high rate of mutation of RNA viruses during replication, a virulent strain may be introduced to domestic poultry as a result of imprecise replication following the introduction of a lowly virulent wild bird strain.

### Risk of introduction to the United States

Potential routes of introduction include live bird transportation [24, 25], fomites [25], poultry product transportation [68], contaminated food and water [69], airborne transmission [70], contaminated vaccines [71, 72], and non-avian carriers [74]. This sub-section focuses specifically on potential routes of introduction into the United States based on import regulations, the presence of wild avian species that have demonstrated a capacity to harbor virulent strains of the pathogen, and the widespread use of live vaccines in conjunction with the inexact replication process of a RNA virus.

#### Legal movement of live birds

Birds are imported into the United States through one of three import centers found in Los Angeles, California; Miami, Florida; and New York, New York. All imported poultry are subject to a 30 day quarantine irrespective of the NDV-status in the country of origin. Imported hatching eggs are differentiated into those derived from NDV-endemic and NDV-free countries and they require a 30 day quarantine or are immediately released following veterinary inspection at the port of entry, respectively.

Import procedures for non-domestic avian species are characterized for: (1) commercial birds—imported for resale, breeding, public display, or any other purpose, excluding pet birds, zoo birds, research birds, or performing, theatrical birds; (2) zoo birds—imported to a zoo facility for breeding, public display, recreational or educational purposes; (3) pet birds (which are further classified as U.S. origin and non-U.S. origin)—imported for personal pleasure of their individual owners and are not intended for resale (paraphrased from the United States Department of Agriculture regulatory guidelines). Regulations for commercial and pet birds (both U.S. origin and non-U.S. origin) are very similar and in both instances an import permit is required along with a health certificate completed by a certified veterinarian in the export country within 30 days of departure. The health certificate must indicate that the bird has not been vaccinated for highly pathogenic avian influenza (HPAI), including H5 and H7 subtypes, may not transit through regions considered to be high risk for HPAI by the Animal and Plant Health Inspection Service (APHIS), must have been vaccinated for NDV more than 21 days prior to export with a lentogenic strain or not be vaccinated (either scenario must be clearly indicated on the health certificate), and must be free of any evidence of communicable diseases. Furthermore, imported commercial and pet birds are subject to a 30-day quarantine at one of the three import centers; pet birds of U.S. origin can, however, complete the quarantine period in the home. Zoo bird importation similarly mandates an import permit and a health certificate stating all of the same information, less one important difference: these birds may be vaccinated for HPAI, including subtypes H5 and H7, but the health certificate must clearly indicate whether or not the bird has been vaccinated. Zoo birds must also undergo a 30-day quarantine period; however, this can occur at either a USDA facility or at an APHIS approved zoo.

Each of the three legal avian import centers in the United States kindly provided data regarding commercial and pet bird importation, specifically information relevant to species imported and country of origin. The Los Angeles import center received 29 164 psittacines between August 2013 and August 2016, the Miami import center received 34 psittacines for commercial or pet use during the 2015 federal fiscal year (October 2014 through September 2015), and the New York import center received 28 psittacines during the 2015 calendar year. Table 2 depicts the types of psittacines that were imported into the U.S. via the three animal import centers. Unspecified psittacines made up a huge majority of the imports (approximately 89%), with parrots and parakeets each accounting for about 5% of imports, and budgerigars, macaws, cockatiels, and cockatoos each representing a tiny proportion of psittacine imports.

**Table 2:** Types of psittacines imported into the U.S. through the three animal import centers—Los Angeles, CA (between August 2013 and August 2016), Miami, FL (during the 2015 federal fiscal year), and New York, NY (during the 2015 calendar year) (*n* = 29 266)

Bird type	Number imported
Unspecified psittacines	25 865
Parrots	1509
Parakeets	1741
Macaws	8
Budgerigars	86
Cockatiels	14
Cockatoos	3
Total	29 226

The continent of origin for the vast majority of psittacines that were imported into the U.S. during the aformentioned time period was Africa, accounting for about 85% of all imports (Table 3). Approximately 11% of psittacine imports were from Australia and 2% of imports were from each Asia and Europe, with few psittacines imported from either North or South America. It is important to note that the country of origin reported is where the birds come from prior to entry in the United States and is not necessarily representative of their true origin.

**Table 3:** Continent of origin for psittacines imported into the U.S. through the three animal import centers—Los Angeles, CA (between August 2013 and August 2016), Miami, FL (during the 2015 federal fiscal year), and New York, NY (during the 2015 calendar year) (*n* = 29 266)

Continent of origin	Number imported
Africa	24 787
Australia	3276
Asia	654
Europe	471
North America	25
South America	13
Total	29 226

#### Legal movement of animal products, byproducts, and animal feed

All avian products and byproducts derived from an NDV-endemic region must be mitigated prior to importation using an APHIS approved method that has been shown to inactivate NDV. Products and byproducts derived from NDV-free regions are allowed to be imported in a raw form. Animal feed containing avian products must be cooked to an internal temperature of 74 °C prior to importation from NDV-endemic regions; however, NDV-free regions may import raw animal feed products.

#### Illegal movement of live birds and their products

**Figure 2:**
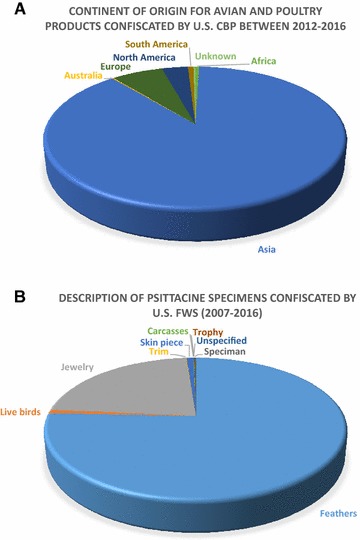
**Country of origin and types of psittacine specimens confiscated by CBP and FWS, respectively. A** A pie chart depicting the continent of origin for the products and specimens confiscated by U.S. Customs and Border Protection between 2012 and 2016 (*n* = 74 837). **B** A pie chart depicting the type of psittacine specimens confiscated by the U.S. Fish and Wildlife Services between 2007 and 2016 (*n* = 4046).

The illegal transport of live animals and their products is of concern for disease transmission because of the lack of governmental oversight, the types of products being moved, and their country of origin and final destination. The U.S. Department of Homeland Security Customs and Border Protection (CBP) is primarily responsible for the confiscation of illegally imported products and specimens from domestic poultry species. Data provided by CBP depicts products and specimens from domestic poultry and birds that were confiscated in the cargo or express courier environment or via international mail facilities. Between calendar years 2012 and 2016, nearly 75 000 products and specimens derived from domestic poultry and other birds were confiscated by CBP. The continent of origin for the majority of products and specimens (~88%) confiscated by CBP is Asia, which is enzootic for virulent NDV. Europe and North America, both of which are free from virulent strains of NDV in domestic poultry, comprise 7 and 3% of the poultry and avian confiscations, respectively. Products and specimens from Africa, Australia, South America, and unknown continent of origin each comprise less than 1% of all confiscations. This data is summarized in Figure 2A.

A large number of samples from NDV susceptible species were confiscated and in many circumstances were derived from continents with regions that are enzootic for NDV. The exact number of products and specimens that are smuggled across the U.S. border is difficult to ascertain and it can be assumed that the products and/or specimens discovered represent a small subsection of the types of goods that are illegally imported into the United States. Because of the types of products confiscated and the regions of the world from which they originate, the illegal importation of domestic poultry and other avian products and specimens pose a risk for virulent NDV introduction.

The United States Fish and Wildlife Service data show that nearly 400 000 wild avian specimens were illegally imported or improperly documented and were subsequently confiscated by the Fish and Wildlife Service between 2007 and 2016 in the United States. Our focus is on psittacines as they have demonstrated their capacity to shed infectious NDV for up to 1 year following infection. Psittacine specimen confiscations accounted for 1% of all the avian contraband confiscated and 75% of those confiscations were feathers, followed by 22% which was jewelry, and live birds, trim, skin pieces, carcasses, unspecified, trophy, and specimens each accounted for less than 1% (Figure 2B). These illegal seizure numbers are concerning as NDV can persist outside of the host, but overall, the risk they pose is likely lower than the risk associated with live birds.

**Figure 3:**
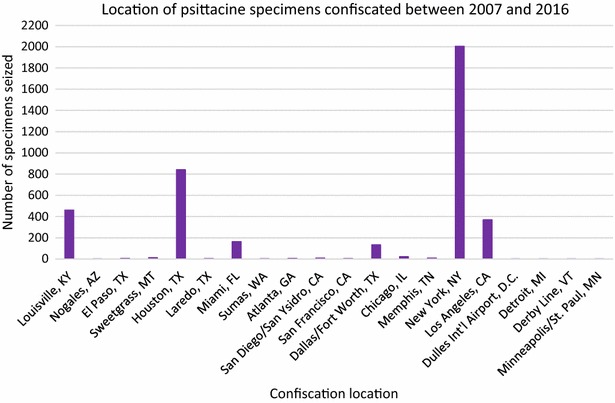
**Psittacine specimens confiscated.** Number of psittacine specimens confiscated by the U.S. Fish and Wildlife Service between 2007 and 2016 at various ports in the United States (*n* = 4046).

Illegally imported psittacine specimens were confiscated at 20 different ports around the country (Figure 3). It is important to note that the graphed data are based on the total number of specimens confiscated, not the number of individual events in which specimens were confiscated. For example, in New York, NY there were three times in which psittacine specimens were confiscated between 2007 and 2016, but one of the incidences involved the confiscation of over 2000 feathers (*n* = 2004).

Estimating the percentage of illegally imported animals that are detected and confiscated in the United States is difficult; however, it is interesting to note the number of avian species, both those that are live birds, as well as feathers and other body parts, which are confiscated. These data suggest that illegal bird and bird product importation could result in an introduction of virulent NDV because of the types of birds being imported, the lack of veterinary oversight and quarantine procedures, and the country of origin (many of which are endemic for virulent strains of NDV).

#### Spillover event from wild avian species

Double-crested cormorants and Columbiform birds, specifically pigeons and doves, have been implicated as amplification or reservoir hosts that appear to be frequently infected with, or carriers of, virulent strains of avian avulavirus [31, 37–41, 43–46]. Spillover events from these wild avian species into domestic poultry operations could have deleterious effects, both in terms of virus-induced morbidity and mortality as well as economic impacts resulting from trade restrictions and the cost of controlling and eradicating the disease [5]. Additionally, substantial economic losses are also associated with less virulent strains as a result of impaired growth, altered feed utilization, and reduced egg production in affected birds [73]. Due to the acute stability of NDV and the apparent overlap between double-crested cormorant breeding and wintering grounds, rock dove habitat, and the majority of clustering relative to domestic poultry operations (Figure 1), a virulent strain harbored by wild avian species could spill over into domestic poultry. The most likely routes of virus introduction would either be a result of fomite transmission (vehicles, equipment, or personnel that become contaminated) or because of direct contamination of domestic poultry or their food or water sources by infected wild species. The latter is a risk primarily associated with pigeons because of their peri-domestic nature and this route of transmission has historically been reported for infection of domestic poultry by infected feces of wild pigeons [25]. The best method to prevent this type of transmission is to have vehicles and equipment that are site-specific or that are thoroughly disinfected between operations, procedures with detailed information on biosecurity procedures following exposure to outside birds, and poultry housing facilities as well as food and water sources that are maintained in a setting that is inaccessible to nuisance creatures.

#### Spontaneous mutation

Avirulent and virulent viruses have varying amino acid sequences at the fusion site which allows for differential cleavage in the host leading to either a localized or systemic infection, respectively. The homology between virulent and avirulent viruses combined with the high mutation rate in RNA viruses make it possible for an avirulent strain to spontaneously mutate into a virulent strain. Only three amino acid substitutions are necessary for this transformation [75].

Between 2007 and 2009, multiple isolates of avian avulavirus were submitted to the National Veterinary Services Laboratory from turkey flocks in the United States and were found to have a fusion cleavage site of 113-K-Q-G-R-F-117 [76]. This sequence contains two of the three necessary amino acids between 113 and 116, to be considered a virulent strain, and a phenylalanine at site 117. Furthermore, these isolates were collected from flocks where the turkeys had signs of mild respiratory disease. Interestingly, pathogenicity tests in embryonated chicken eggs and day old chicks found these viruses to be low virulence in chickens and are likely host-adapted to turkeys. This finding however, is of concern as repeated viral passages through infected birds elevates the probability of a spontaneous mutation into a virulent strain.

## Conclusions

Virulent Newcastle disease virus, represented by various strains of avian avulavirus, is a highly transmissible virus that results in near-uniform mortality in domestic poultry species. Furthermore, detection of virulent NDV is a mandatory reportable disease to OIE and results in severe, and often long-lasting, restrictions in international trade. Economic losses associated with the disease extend well beyond trade embargos, including eradication programs aimed at culling birds from infected farms and preventing further transmission between flocks. Double-crested cormorants and rock doves have been implicated as a potential reservoir species in the United States, in addition to vaccinated domestic poultry species. Newcastle disease is a zoonotic disease; however, a huge majority of individuals who become infected are asymptomatic or develop a mild, self-limiting disease. A plethora of diagnostic tools exist, ranging from virus isolation followed by pathotyping, to RNA detection, and serological methods. Continuing to closely monitor both domestic poultry as well as wild avian species is crucial in order to detect an outbreak quickly and eradicate an exotic introduction swiftly. This surveillance necessitates reliable diagnostic methodologies and preferably tools that can detect multiple pathogens simultaneously using metagenomics capabilities. Dimitrov et al. describe a next-generation sequencing approach for the characterization of RNA virus genomes which allows for efficient, cost-effective diagnostics [77]. Practicing robust biosecurity measures, especially those related to preventing pigeons from accessing food and water sources of domestic poultry, are essential for avoiding a disease outbreak.

Psittacines are often considered to be higher risk species as they can shed NDV for up to a year; however, the legal route of importation of these birds, and all others, into the United States is quite comprehensive. All legally imported birds are quarantined for 30 days during which time they are tested for NDV using the virus isolation assay and upon completion of the quarantine period and a negative NDV test, are released. Illegally imported birds that are confiscated are either euthanized or quarantined and tested prior to release, depending on oversight from both USDA Veterinary Services and U.S. Fish and Wildlife Service. The strict import regulations for live birds and their products, as well as stringent guidelines related to NDV as a Tier 1 Select Agent, are of high importance as human activities have been shown to result in spillover events [78]. The illegal importation of birds and their products is difficult to control, manage, or regulate. This route certainly serves as a risk for NDV importation and subsequently, introduction.

The wild bird reservoirs of NDV found in the U.S. complicate the potential for viral incursion in domestic poultry. Understanding how these moderately and highly virulent viruses are maintained in pigeons and cormorants is fundamental to our comprehension of the landscape and other risk factors that substantially contribute to a viral incursion in domestic poultry. A surveillance program designed to sample birds of the Columbidae family could identify avian avulavirus strains circulating in wild birds and could also be used to assess the spillover of vaccines used in domestic poultry into wild bird populations. Additionally, sampling confiscated psittacine specimens that were illegally imported could provide increased understanding of the types of viruses that are entering the U.S. and may lead to prophylactic measures to protect poultry in the U.S. Backyard poultry flocks are believed to present a risk of viral amplification in the event of an NDV introduction as they are typically unvaccinated and extensively reared. NDV in backyard flocks that spilled over to commercial poultry operations was linked to the most recent outbreak in the United States and yields an ever-present challenge. The discovery of virulent strains of NDV circulating in backyard chickens in Bulgaria and Ukraine between 2002 and 2013 suggests a domestic or urban cycle of viral maintenance [69]. Maintaining robust surveillance and reporting systems is crucial in order to detect and control a foreign animal disease outbreak, especially in an at-risk population, such as backyard poultry.

## Additional file

**Additional file 1.** **Summary of temporal and geographical distribution of NDV strains.** Data regarding class and genotype, strain virulence, and global distribution are based on Dimitrov et al. [27], with additional information from other manuscripts (cited in table).
