# Correction to: A review of virulent Newcastle disease viruses in the United States and the role of wild birds in viral persistence and spread

**DOI:** 10.1186/s13567-017-0485-7

**Published:** 2017-11-16

**Authors:** Vienna R. Brown, Sarah N. Bevins

**Affiliations:** 1 Oak Ridge Institute for Science and Education (ORISE) supported by the U.S. Department of Homeland Security (DHS), Science and Technology Directorate (S&T), Chemical and Biological Defense Division (CBD), Oak Ridge, TN USA; 2 United States Department of Agriculture, Animal and Plant Health Inspection Service Wildlife Services, National Wildlife Research Center, Fort Collins, CO USA

## Correction to: Vet Res (2017) 48:68 10.1186/s13567-017-0475-9

After publication of the article [1], it has been brought to our attention that Newcastle disease virus was incorrectly labeled as a Tier 1 USDA Select Agent. Newcastle disease virus is a USDA Select Agent but it is not a Tier 1 agent.
