# Supplementary material for: A review of virulent Newcastle disease viruses in the United States and the role of wild birds in viral persistence and spread
Source: Vet Res. 2017 Oct 26;48:68. doi: 10.1186/s13567-017-0475-9 (PMC5659000; doi:10.1186/s13567-017-0475-9)
Supplement: Supplementary file 1 — Additional file 1. Summary of temporal and geographical distribution of NDV strains. Data regarding class and genotype, strain virulence, and global distribution are based on Dimitrov et al. [27], with additional information from other manuscripts (cited in table). [file 13567_2017_475_MOESM1_ESM.docx]

| **Class** | **Genotype** | **Sub-genotypes** | **Virulence** | **Global distribution** |
| --- | --- | --- | --- | --- |
| I |  | 1a | Lentogenic | Recovered from domestic ducks, chickens and geese in China during 2007-2010 |
|  |  | 1b | Lentogenic | Isolated from chickens, ducks, black swans, peafowl, egrets, and herons in China during 2009 and 2013 |
|  |  | 1c | Lentogenic | Isolated from ducks, swans, geese, and shorebirds in the U.S. since 1998; isolated in Europe (1999-2010) and Asia from a variety of wild waterbirds as well as domestic ducks in China |
| II | I | Ia | Lentogenic  Velogenic | Isolated from wild waterfowl and poultry samples in China, Columbia, Malaysia, and S. Korea in 1998-2014  Recovered from Australia in chickens between 1998 and 2002 |
|  |  | Ib | Lentogenic | Recovered from a variety of wild and domestic waterfowl in China, Japan, Luxembourg, Madagascar, Nigeria, Russia, S. Korea, Ukraine, and the U.S. between 2001 and 2011 |
|  |  | Ic | Lentogenic | Isolated from shorebirds, waterfowl, gulls and various landbirds in Japan, Mexico, Russia, Sweden, and the U.S. between 1994 and 2009 |
|  | II |  | Lentogenic and velogenic | Historically 50% velogens, now only virulent II NDV’s are from China, Egypt, and India and are likely due to lab contamination or releases [70] |
|  | III |  | Mesogenic | Isolated from Australia in 1932, Japan between 1930-1960, sporadic isolation in the 1960s and 1990s in the U.K., Taiwan, Zimbabwe, and Singapore; recovered from chickens in Pakistan in 1974 and from chickens and domestic waterfowl in China between 2000-2005 |
|  | IV |  | Velogenic | Recovered from poultry in Europe between 1933-1944, the predominant viruses isolated in Europe before 1970; found in poultry in Africa in 1991, pigeons in Asia in 1989, and poultry in Russia in 1970 |
|  | V | Va | Mesogenic and velogenic | Viruses recovered from the U.S. and Canada since 1995 in Double-crested Cormorants [40] |
|  |  | Vb | Velogenic | Recovered from poultry, caged-birds, and peri-domestic species in the U.S., Brazil, C. America, and Africa since the 1970s |
|  |  | Vc | Velogenic | Circulating in Mexico and C. America mostly in poultry but also caged-birds, tree-ducks, and quail |
|  |  | Vd | Velogenic | Isolated from poultry in Kenya and Uganda during 2010-2011 |
|  | VI | Via | Mesogenic and velogenic | Reported primarily in Columbidae birds in Asia, Europe, the Middle East, and the U.S. since the 1990s |
|  |  | Vib | Mesogenic and velogenic | Isolated from pigeons in Argentina, China, Italy, and the U.S. between 1984 and 2007 |
|  |  | Vic | Velogenic | Recovered from chickens in East Asia in the 1980s and 1990s |
|  |  | Vid | Velogenic | Found in poultry in Europe during the 1990s |
|  |  | Vie | Velogenic | Recovered from Columbidae birds (pigeons) in China from 1996-2012 |
|  |  | Vif | Velogenic | Recovered from Columbidae birds (pigeons) in the U.S. from 1984-2007 |
|  |  | Vig | Velogenic | Recovered from Columbidae birds (pigeons, doves) in Kenya and Nigeria from 2007-2013 |
|  |  | Vih | Velogenic | Recovered from a pigeon in Argentina in 1997 |
|  |  | Vii | Velogenic | Recovered from Columbidae birds (collared doves) in Italy from 2010-2011 |
|  | VII | VIIa | Velogenic | Isolates recovered from poultry in Western Europe in the 1990s |
|  |  | VIIb | Velogenic | Recovered from gallinaceous poultry and domestic waterfowl in China during 1998-2014, Vietnam in 2007, and Israel in 2011-2014; associated with outbreaks in Europe, Turkey, S. Africa, Mozambique, Kazakhstan, the Far East, the Middle East, and India in the 1990s and early 2000s |
|  |  | VIIc | Velogenic | Isolated from chickens and pigeons from China and Taiwan between 1996-2000, sporadically identified in poultry in Europe during 1996-1997 |
|  |  | VIId | Velogenic | Recovered from China in 1998-2013, S. Korea in 2000-2005, and Columbia in 2006-2010; collected in infrequently from poultry in Israel, S. Africa, Ukraine, and Venezuela between 2004-2009; found in wild birds with clinical disease in China and Serbia in 2006-2007 |
|  |  | VIIe | Velogenic | Identified in chickens and domestic waterfowl in China, Japan, Taiwan, and Vietnam during 1997-2014 |
|  |  | VIIf | Velogenic | Recovered from domestic poultry and pigeons in China during 1996-2008 |
|  |  | VIIg | Velogenic | Believed to represent recombinant strains |
|  |  | VIIh | Velogenic | Originated from chickens in Bali, Indonesia, and Malaysia in 2007-2011 |
|  |  | VIIi | Velogenic | Isolated primarily from chickens in Indonesia, Israel, and Pakistan during 2010-2013, as well as Bulgaria and Hungary [71] |
|  | VIII |  | Velogenic | Found in chickens in Argentina, China and Malaysia in the 1960s to the 1980s |
|  | IX |  | Velogenic | Originally recovered in China in the 1940s, isolates discovered in China between 1985-2011, mostly in domestic poultry but virus has been isolated from asymptomatic wild birds |
|  | X |  | Lentogenic | Isolated from wild waterfowl in the U.S. and Argentina between 1986 and 2004, also found in Minnesota and Wisconsin in commercial turkeys |
|  | XI |  | Velogenic | Recovered from chickens in Madagascar during 2008-2011 |
|  | XII |  | Velogenic | Identified in chickens in S. America in 2008-2009 and geese in China during 2010-2011 |
|  | XIII | XIIIa | Velogenic | Recovered from chickens in Europe in 1997, Asia during 1997-2010, Africa in 1995-2008, the Middle East in 2008-2011, and Sweden in 2016 |
|  |  | XIIIb | Velogenic | Found in India and Pakistan during 2003-2013 |
|  | XIV | XIVa | Velogenic | Recovered from chickens and turkeys in Niger and Nigeria during 2006-2011 |
|  |  | XIVb | Velogenic | Isolated from samples collected from chickens, turkeys, and guinea fowl in Nigeria in 2007-2011 |
|  | XV |  | Velogenic | Represent recombinant strains isolated from chickens and geese in China between 1997 and 2004 |
|  | XVI |  | Velogenic | Identified from isolates recovered from chickens in Mexico in 1947 and the Dominican Republic during 1986-2008 |
|  | XVII | XVIIa | Velogenic | Recovered from chickens in Benin, Burkina Faso, Cameroon, Ivory Coast, Mali, Niger, and Nigeria from 2006-2011 |
|  |  | XVIIb | Velogenic | Originated from chickens in Nigeria during 2006-2011 |
|  | XVIII | XVIIIa | Velogenic | Recovered from chickens and guinea fowl in the Ivory Coast, Mali, and Mauritania during 2006-2010 |
|  |  | XVIIIb | Velogenic | Isolated from poultry in the Ivory Coast, Mali, Nigeria, and Togo |
